# The Foulage Test: Proof of Concept of a Novel Stepping Test Using a Stabilometer

**DOI:** 10.7759/cureus.24763

**Published:** 2022-05-05

**Authors:** Toru Miwa, Tomohisa Yasuda, Kishiko Sunami, Takanobu Kunihiro, Kouichi Yasuda

**Affiliations:** 1 Otolaryngology-Head and Neck Surgery, Tazuke Kofukai Medical Research Institute, Kitano Hospital, Osaka, JPN; 2 Otolaryngology, Yasuda ENT Clinic, Tokyo, JPN; 3 Otolaryngology-Head and Neck Surgery, Osaka City University, Graduate School of Medicine, Osaka, JPN; 4 Otolaryngology, International University of Health and Welfare, Atami, JPN

**Keywords:** stabilometer, body balance, dynamic equilibrium, stepping test, foulage test

## Abstract

Objective

In this article, we aimed to describe the Foulage test (FT) and investigate the test-retest reliability of parameters recorded during stepping execution in healthy adults.

Materials and methods

This was a single-center prospective cohort study conducted at an outpatient clinic. It included five healthy male participants [mean age ± standard deviation (SD): 27 ± 5.4 years]. The FT was performed first with the participants’ eyes open and again with their eyes closed. If the heel height was not within 2-6 cm, the participant was asked to restart. The FT value and variance of steps were automatically calculated. To verify the influence of heel height, measurements were taken at different heel heights. We also evaluated the Romberg ratio (calculated from the parameters with eyes open and closed) and defined it as the dynamic Romberg ratio. Correlations between parameters were also assessed.

Results

The parameters’ FT value (front-back width of the band of locus shape) and variance of steps plateaued under stable conditions within a heel height of 2-6 cm. FT values and variance of steps were strongly correlated. The dynamic Romberg ratios by FT value and by the variance of steps were also strongly correlated.

Conclusions

The FT is a dynamic and reproducible equilibrium function test that can quantify agitation with the eyes open or closed in general outpatient clinics, and it may be employed as a clinically useful method for the observation of clinical courses in patients with vestibular disorders.

## Introduction

To date, static and dynamic postural controls have been studied using various methods [1-6]. However, these examinations have several limitations as adequate balance tests. For example, the Fukuda stepping test cannot quantitate the parameters of body balance function [4]. Recently, we conducted pilot studies of the Foulage test (FT), a novel non-invasive quantitative stepping method in which only the heels are alternately raised while the balls of both feet remain in contact with the ground [7-13]. “Foulage” is a French word for “grape trampling,” a process of winemaking wherein grapes are crushed with bare feet. This word is used because the ground-contact method in grape trampling is different from the conventional stepping method, in which the sole of the foot is raised. Our FT excludes the participants’ free stepping movement as a variable by eliminating active and agitation elements derived from the varied height of the legs.

In this study, we introduce the methods of the FT and investigate the repeatability (test-retest reliability) of parameters recorded during stepping execution in healthy adults as well as the number of trials required to produce repeatable results. We hypothesized that this test would provide new possibilities for clinic-based evaluation of vestibular disorders compared with the traditional posturography or stabilometry tests.

## Materials and methods

Participants

The present protocol was developed for use in humans. To analyze adequate test conditions, we recruited five healthy men [age range: 22-34 years; mean age ± standard deviation (SD): 27 ± 5.4 years]. The study adhered to the tenets of the Declaration of Helsinki and was approved by the Institutional Ethics Committee of Yasuda ENT clinic (approval number: 105). All participants provided written informed consent.

Procedure

**Figure 1:**
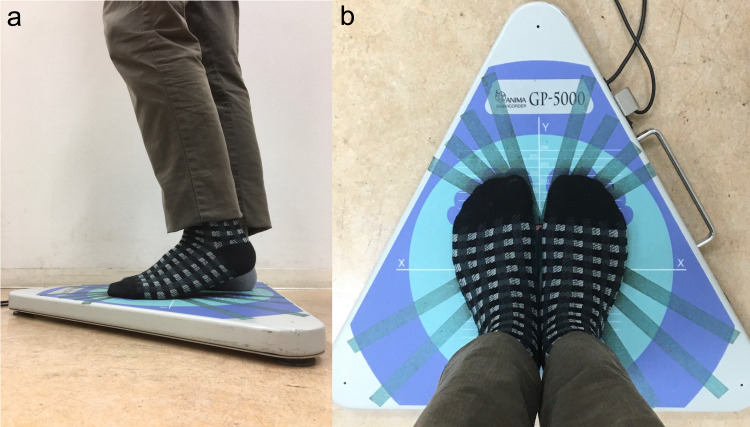
An example of the conditions maintained during the Foulage test (a) Lifting only the heels. (b) Feet kept flat

Before starting the test, participants were instructed to stand with their feet together (straight and parallel to each other), lifting only the heels alternately at a height of 2-6 cm in tune with a metronome beat at a tempo of 120 bpm. Heel height was measured by instructors visually within 2-6 cm. Next, the participants were asked to step on the center of a stabilometer (GP5000; Anima, Tokyo, Japan) with their feet together, lifting only the heels alternately at the same tempo, for 60 seconds with their eyes open (Figure 1). The same procedure was repeated with their eyes closed. Participants were observed carefully to prevent falls. If the heel height was not within 2-6 cm, an error message was shown and the participant was asked to restart.

The FT value (described below) and variance of steps were automatically calculated, and graphs were drawn using the FT software and Microsoft Excel (Microsoft Corporation, Redmond, CA), respectively (data available on request).

The step-by-step protocol is available from protocols.io as a data review (https://www.protocols.io/view/foulage-test-bvymn7u6) [14].

Program code

Sampling

The sampling frequency was 20 Hz for 60 seconds. The 1200 plots of the center of pressure (COP) with the eyes open and closed were input into an Excel file.

Selecting the Values of the Right and Left Steps

The right step was the maximum x-axis value per second, and the left step was the minimum x-axis value per second, as shown in the following equations.

$\mathit{Right\ steps=MAX1\ (0-1),MAX2 (2-3),MAX3 (4-5)...}$

$\mathit{Left\ steps=MIN1(0-1), MIN2(2-3), MIN3(4-5)... }$

Integration of Right and Left Steps

The center point was the mean of 60 right steps and 60 left steps. Both steps were integrated, and the mean was calculated.

$\mathit{Both\ steps=MEAN\ \left(MAX1+MIN1+MAX2+MIN2+MAX3+MIN3\ldots\right)}$

Calculation of the Variance of Steps

The variance of steps was calculated by determining the SD of 120 steps using the following formula:

$\mathit{Variance\ of\ steps=STDEV\ {MAX1\ (MEAN-MIN1),\ MAX2\ (MEAN-MIN2),\ MAX3\ (MEAN-MIN3)...}}$

Parameters

FT Value

**Figure 2:**
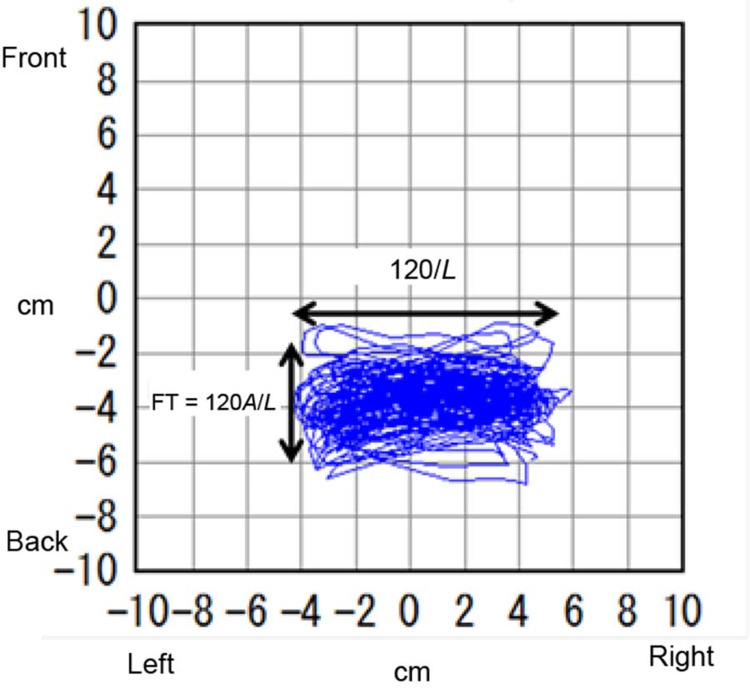
Results and Foulage test (FT) value with the eyes open The FT value is equivalent to the front-back width of the band of locus shape. FT = 120A/L, where A is the surrounding area and L is the total locus length

The surrounding area (A) and total locus length (L) were recorded using a stabilometer. The mean distance of the steps, one step’s locus length, is L divided by 120. The FT value is the surrounding area (A) divided by the mean distance of the steps (Figure 2). The FT value is equivalent to the front-back width of the band of the locus shape.

$FT\ value=A/(L/120)=120\ A/L$

Variance of Steps

**Figure 3:**
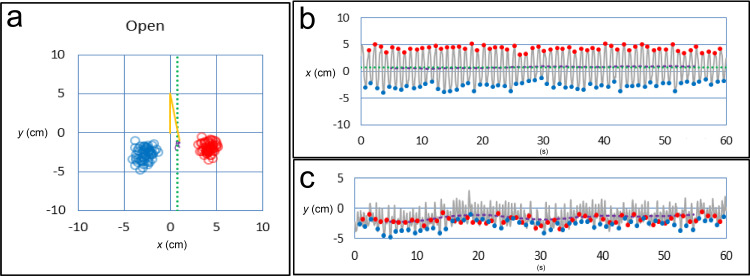
Variance of steps with eyes open (a) Variance of steps. (b) Standard deviation of the x-axis. (c) Standard deviation of the y-axis. Blue points, left-heel steps; red points, right-heel steps

Sampling data at 20 Hz resulted in the measurement of COP coordinates every 0.05 seconds. The point when the COP turns from the right to the left side is the step of the right heel, whereas the point when the COP turns from the left to the right side is the left step. The absolute values of right and left COP lengths (x- and y-axes) were obtained, and the mean and SD (x- and y-axes) were calculated. The SD of the x-coordinate was the variance of steps in the right-left direction, and the SD of the y-coordinate was the variance in the front-back direction. The total variance in two dimensions was calculated as x - SD multiplied by y - SD. We plotted the steps, right-left locus (red), and front-back locus (blue) in a time graph (Figure 3).

$x-SD=\sqrt{\frac{1}{60}\sum_{i=1}^{60}\left(x_i-\bar{x}\right)^2},\ y-SD=\sqrt{\frac{1}{60}\sum_{i=1}^{60}\left(y_i-\bar{y}\right)^2}$

$Variance\ of\ steps=(x-SD)\times(y-SD)$

Dynamic Romberg ratio

The FT makes the calculation of the Romberg ratio possible [10,12,15] as testing is performed in both open- and closed-eye conditions. We defined the Romberg ratio in FT parameters as the “dynamic Romberg ratio.”

$\mathit{Dynamic\ Romberg\ ratio\ by\ FT\ value=\frac{120\ A/L\ with\ eyes\ closed}{120\ A/L\ with\ eyes\ open}\ }$

$Dynamic\ Romberg\ ratio\ by\ variance\ of\ steps=\frac{\left(x-SD\right)\times\left(y-SD\right)with\ eyes\ closed}{\left(x-SD\right)\times\left(y-SD\right)\ with\ eyes\ open}$

Analysis of adequate conditions

To verify the influence of heel height on each parameter, recordings were performed five times at heel heights of 1, 2, 4, 6, and 15 cm.

Statistical analysis

The FT program ran automatically after the data were acquired. To determine the accepted degrees of heel height, Pearson’s linear correlation analysis was performed. Statistical significance was set at a p-value of <0.05. All statistical analyses were performed using GraphPad Prism version 8.0.0 for Windows (GraphPad Software, San Diego, CA).

## Results

Calculation of parameters

**Figure 4:**
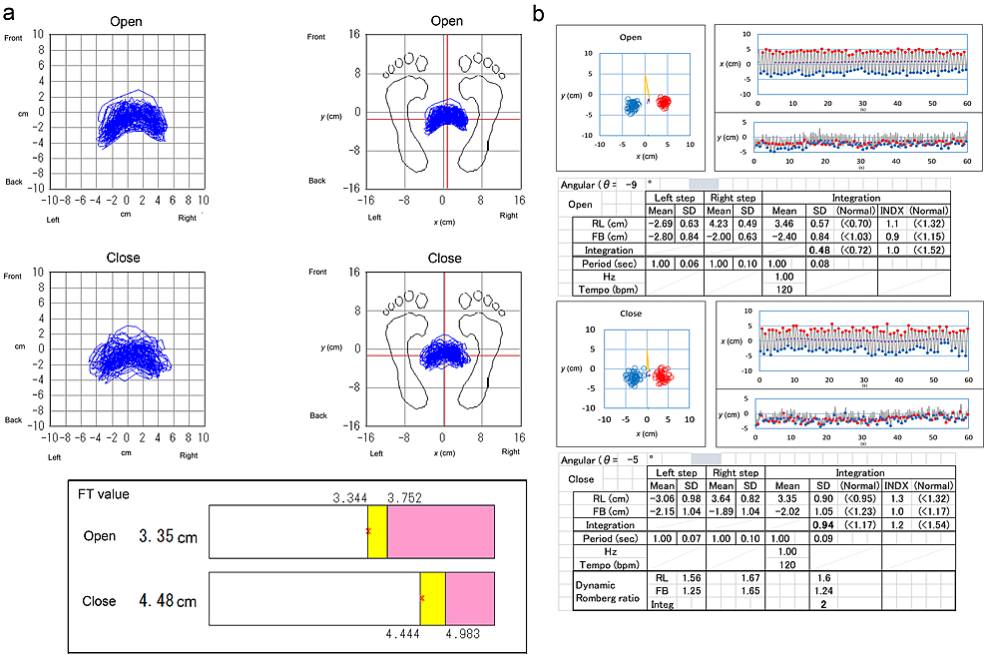
Drawings and results (a) Step drawing and Foulage test (FT) value results. Blue: locus of the center of pressure; in the bar graph of the panel (a), red asterisks: FT value; yellow bands: para-abnormal area; pink bands: the abnormal area defined in another study [1] (data published in Japanese). (b) Drawing, the variance of step results, and dynamic Romberg ratio. Blue: left steps; red: right steps FT: Foulage test; SD: standard deviation; RL: right to left; FB: front to back

FT values with eyes open and closed were 3.35 and 4.48 cm, respectively (Figure 4). The variance of steps with eyes open and closed was 0.48 and 0.94, respectively (Figure 4b). The dynamic Romberg ratios by FT value and variance of steps were 1.34 and 1.99, respectively (Figure 4b).

Analysis of adequate conditions

**Figure 5:**
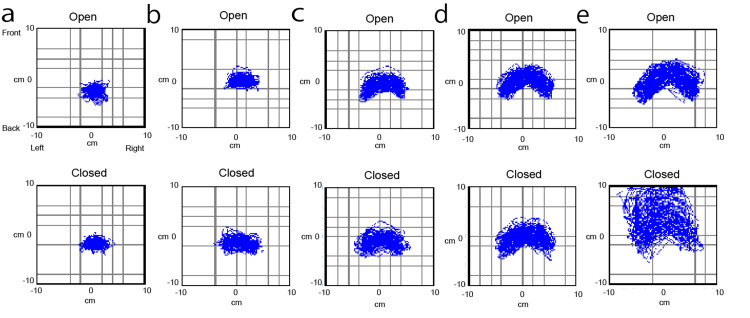
Locus of different heel heights (a) 1 cm, the locus with almost no heel lift was extremely small, close to a static state; (b) 2 cm; (c) 4 cm; (d) 6 cm, as the heel was lifted higher, the locus extended toward the left and right directions in a band shape and bent in an inverted V-shape that was convex forward; (e) at 15 cm, turbulence occurred, and the front-back width tended to increase, especially with closed eyes

The typical five-stage representative results with different heel heights are shown in Figure 5. At a 1-cm heel height, the locus became a small circle (Figure 5a). As the heel height increased, the locus extended toward the left and right directions, forming a bar shape (Figures 5b-5c) and bent in an inverted V-shape that was convex forward (Figure 5d). When the heel height was further raised, turbulence occurred, and the front-back width tended to increase, especially with closed eyes (Figure 5e).

**Figure 6:**
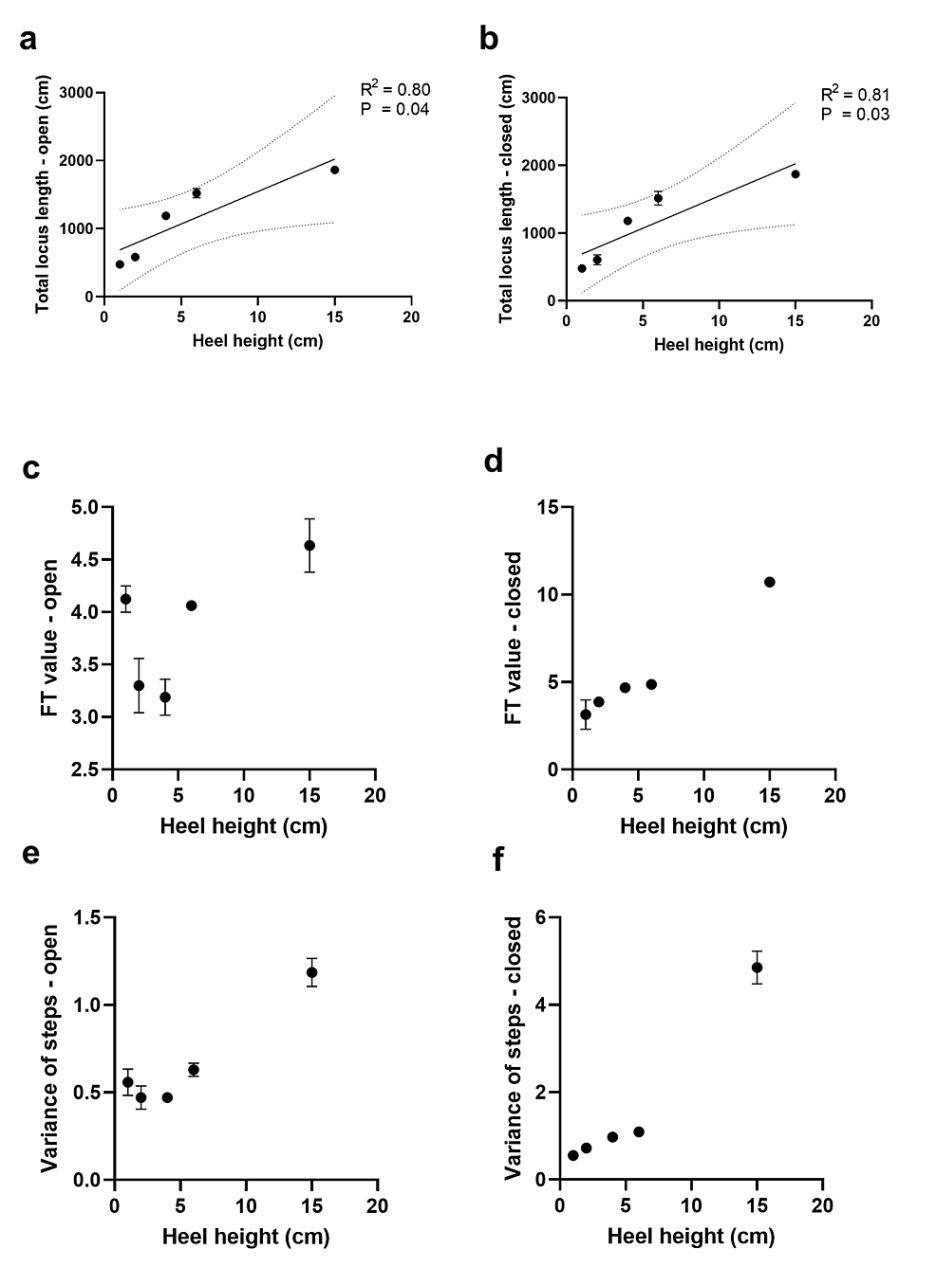
Correlation between heel height and total locus lengths, Foulage test (FT) values, and variance of steps (a, b) The mean total locus lengths increased with the increasing heel height and were strongly linearly correlated with eyes open (a) and closed (b). (c–f) Correlation between heel height and mean FT value with eyes open (c), mean FT value with eyes closed (d), the mean variance of steps with eyes open (e), and the mean variance of steps with eyes closed (f). In the stable range of stepping, the mean FT value and variance of steps were almost constant, except for the FT value with eyes open because of cognition by watching around in participants. At 15 cm, variance increased dramatically. Straight solid lines: linear correlation; dotted lines: 95% confidence intervals; bars: standard deviation

The mean total locus lengths increased with the increasing heel height and were strongly linearly correlated with eyes open (R^2^=0.80, p=0.04; Figure 6a) and closed (R^2^=0.81, p=0.03; Figure 6b). In the stable stepping range, the mean FT value or variance of steps plateaued with increasing heel height (Figures 6c-6f). While the stepping was stable, the movement of the COP maintained regularity and repetitiveness (at 2-6 cm); however, when it was raised to an extremely high level (at 15 cm), turbulence occurred, and the mean FT value and variance of steps tended to increase (Figures 6c-6f). Therefore, we considered that the adequate conditions of heel height were 2-6 cm because the steps maintained regularity and repetitiveness.

**Figure 7:**
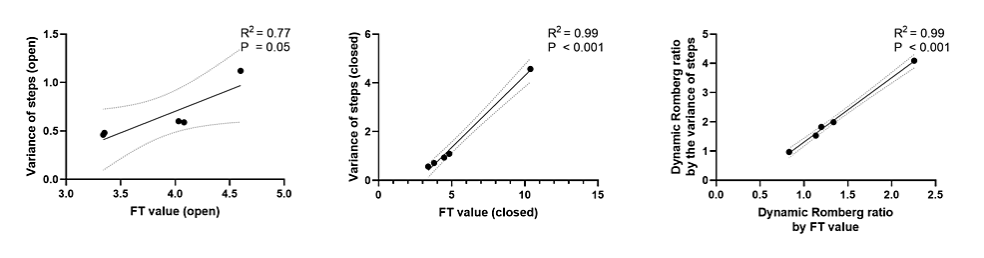
Correlation between Foulage test (FT) value and variance of steps (a, b) FT value and variance of steps were positively correlated within 6 cm of heel height with eyes open (a) and closed (b). (c) The dynamic Romberg ratio by the FT value and variance of steps were positively correlated. Straight solid lines: linear correlation; dotted lines: 95% confidence intervals

Furthermore, there was a strong correlation between FT value and variance of steps (open R^2^=0.77, p=0.05; Figure 7a; closed R^2^=0.99, p<0.001; Figure 7b). In addition, there was a significant correlation between the dynamic Romberg ratio by FT value and that by the variance of steps (R^2^=0.99, p<0.001; Figure 7c).

## Discussion

We conducted a pilot study of the FT, a novel non-invasive quantitative stepping method wherein only the heels are alternately raised while the balls of both feet remain in contact with the ground. In the stable stepping of a healthy person, the left and right folds and the front-back shifts are constant to a certain extent; 120 steps and 60 reciprocations with a certain width are added to the same locus, which is an inverted V-shaped locus. The inverted V-shaped locus is close to a rectangle, bent at the center, and its long side can be considered as the average moving distance (L/120) of one step. The outer peripheral surrounding area of the inverted V-shaped locus (i.e., the area, A, of the bent rectangle) can be estimated by multiplying the length of the long and short sides (front-back width). We defined front-back width as the FT value and the variance of the steps as the instability parameter. In addition, the dynamic Romberg ratio can estimate the cause of vertigo/dizziness, whether from a peripheral vestibular disorder or a central vestibular disorder [7-13].

Postural control is the foundation of our ability to stand and walk independently. Deterioration in postural control due to normal aging or vestibular diseases, such as Meniere's disease, benign paroxysmal positional vertigo, and vestibular neuritis, is associated with an increased risk of falls incurred during daily activities [14]. Currently, the most common method of evaluating postural control in the clinic is to perform a force plate analysis of the COP displacement during quiet stances [1-3]. However, force plate-based posturography and stabilometry do not reflect the condition of patients because they are performed in static standing conditions. Therefore, dynamic equilibrium tests, including stepping [4], walking [3], and timed-up-and-go tests [5], are conducted in the clinic. For the quantitative analysis of dynamic equilibrium, an optical three-dimensional motion capture system for gait analysis has been developed; however, it is not widely used because of installation space requirements and high cost [6]. In addition, assessment parameters have not yet been fully established. Previous studies have shown the limitations of a stepping test without any precise instructions to the participant regarding the motion, implying that more precise instructions during the test would increase the reliability [4,15]. The FT can eliminate the rough motion of the knees during the test because the balls of both feet remain in contact with the ground, and step timing is controlled by a metronome. Therefore, our methods do not need instructions regarding motions. However, our FT has a few limitations. Firstly, the FT cannot diagnose specific diseases by indicating the motion of the COP during stepping. Second, this is a proof-of-concept study and has not yet been used to examine patients with vestibular disorders. Prospective multicenter cohort studies are needed to further clarify the clinical usefulness of the FT.

## Conclusions

The FT is a dynamic equilibrium function test that can quantify agitation with eyes open or closed in general outpatient clinics and it may be clinically useful. Our methods can evaluate stepping motion more accurately because the rough motion is restricted. We anticipate that this test will present new possibilities for the future of the traditional posturography or stabilometry tests.
